# Verbal autopsy completion rate and factors associated with undetermined cause of death in a rural resource-poor setting of Tanzania

**DOI:** 10.1186/1478-7954-9-41

**Published:** 2011-08-05

**Authors:** Mathew A Mwanyangala, Honorathy M Urassa, Jensen C Rutashobya, Chrisostom C Mahutanga, Angelina M Lutambi, Deodatus V Maliti, Honorati M Masanja, Salim K Abdulla, Rose N Lema

**Affiliations:** 1 Ifakara Health Institute, Off passage, P.o.Box 53, Off Mlabani, Ifakara, Kilombero, Morogoro, Tanzania; 2 Ifakara Health Institute, Plot 463, Kiko Avenue, Mikocheni, P.o.Box 78373, Dar es Salaam, Tanzania

**Keywords:** Verbal autopsy (VA), completion rate, undetermined, HDSS

## Abstract

**Background:**

Verbal autopsy (VA) is a widely used tool to assign probable cause of death in areas with inadequate vital registration systems. Its uses in priority setting and health planning are well documented in sub-Saharan Africa (SSA) and Asia. However, there is a lack of data related to VA processing and completion rates in assigning causes of death in a community. There is also a lack of data on factors associated with undetermined causes of death documented in SSA. There is a need for such information for understanding the gaps in VA processing and better estimating disease burden.

**Objective:**

The study's intent was to determine the completion rate of VA and factors associated with assigning undetermined causes of death in rural Tanzania.

**Methods:**

A database of deaths reported from the Ifakara Health and Demographic Surveillance System from 2002 to 2007 was used. Completion rates were determined at the following stages of processing: 1) death identified; 2) VA interviews conducted; 3) VA forms submitted to physicians; 4) coding and assigning of cause of death. Logistic regression was used to determine factors associated with deaths coded as "undetermined."

**Results:**

The completion rate of VA after identification of death and the VA interview ranged from 83% in 2002 and 89% in 2007. Ninety-four percent of deaths submitted to physicians were assigned a specific cause, with 31% of the causes coded as undetermined. Neonates and child deaths that occurred outside health facilities were associated with a high rate of undetermined classification (33%, odds ratio [OR] = 1.33, 95% confidence interval [CI] (1.05, 1.67), p = 0.016). Respondents reporting high education levels were less likely to be associated with deaths that were classified as undetermined (24%, OR = 0.76, 95% CI (0.60, -0.96), p = 0.023). Being a child of the deceased compared to a partner (husband or wife) was more likely to be associated with undetermined cause of death classification (OR = 1.35, 95% CI (1.04, 1.75), p = 0.023).

**Conclusion:**

Every year, there is a high completion rate of VA in the initial stages of processing; however, a number of VAs are lost during the processing. Most of the losses occur at the final step, physicians' determination of cause of death. The type of respondent and place of death had a significant effect on final determination of the plausible cause of death. The finding provides some insight into the factors affecting full coverage of verbal autopsy diagnosis and the limitations of causes of death based on VA in SSA. Although physician review is the most commonly used method in ascertaining probable cause of death, we suggest further work needs to be done to address the challenges faced by physicians in interpreting VA forms. There is need for an alternative to or improvement of the methods of physician review.

## Background

Verbal autopsy (VA) is a commonly used tool to ascertain causes of death. In many developing countries, cause of death data are limited because most deaths take place outside health facilities [1]. In addition, in some countries, vital statistics from vital registration systems are incomplete or do not exist. As a result, VA is often necessary for cause of death data determination [2-4] and results from VA are widely used for health planning, priority setting, monitoring, and evaluations [5-7]. In sub-Saharan Africa (SSA) and Asia, VA is used to obtain estimates on the distribution of causes of death and has become a routinely used tool to provide information on the burden of the disease [5,8-10]. VA has been shown to provide the best results to obtain the specific causes of death in most of SSA [11]. In order to play this potential role, VA methodology needs to be generalizable and responsive to community needs.

VA is a process involving completion of death identifications, VA interviews, and cause of death ascertainment. VA is based on the premises that the primary caregiver, usually a family member, can recall, volunteer, and recognize symptoms experienced by the deceased that can be interpreted later to derive a probable cause of death. Several studies have documented challenges with the process of the interview in terms of interviewers, respondents, recall period, and language [12-15]. There have been problems with questionnaires, such as grouping and comprehensiveness of VA forms, closed- versus open-ended questions, and linguistic appropriateness [16-19]. Another overarching issue is the diversity in VA questionnaires used across different countries; although, recently, there has been great effort internationally to harmonize these tools [13-16]. Also, there are different methods for interpreting VA data to derive the probable causes of death including physician review, algorithms, probabilistic methods, and use of artificial neural networks.

The process of VA includes identification of deaths in the community, documentation of the event [20-22], and interview of the caretaker of the deceased person. However, not all reported deaths result in interviews or specific assignment of causes of death. There has been limited systematic documentation of the completion rates at each step of the VA process, ending when a cause of death is assigned. The current study set out to determine the completion rates of the VA process and factors associated with failure to assign a cause of death.

It is important to understand the gaps in current VA methods and explore how improve them [17]. Such information is needed for optimal design of VA tools that will enable better estimates of disease burden and understanding of the limitations of VA questionnaire administration from the stage of identifying a death to the end point of assigning a cause of death. Better understanding of the VA process will contribute in decision-making on whether to use physician review, algorithms, artificial neural networks, or probabilistic methods to interpret and assign causes of death to estimate cause-specific mortality in rural settings of Tanzania.

## Methods

### Study area

The Ifakara Health and Demographic Surveillance System (HDSS) is a part of the INDEPTH Network http://www.indepth-network.org. It was established in 1996, and since January 1997, all individuals are followed through households visits once in every four months. The surveillance area covers a total of 2,400 km^2 ^of Guinea savanna in the floodplain of the Kilombero River, which divides the two districts of Kilombero and Ulanga in the Morogoro region. During the household visit the field interviewer updates and records basic demographic events including deaths, birth, pregnancy, and migration. Since, 2002 all deaths reported were followed with VA in order to ascertain the possible cause of death.

### Death identification

The HDSS field interviewers identify and register deaths during the routine household visit. During that visit, the interviewer informs the respondent that within a specified period of time another person will make a visit to document details about the death. Each death is recorded in forms that are collected, as well as in household register books. These forms are submitted to a data clerk for logging and to data management for entry in the database. The lists of deaths per VA supervisor zone are presented with basic demographic and household information in order to facilitate finding the residency of the deceased.

### The VA tool

The VA is a postmortem in-depth interview with the primary caregivers of the deceased [17]. VA questionnaires are structured into sections, including background, short narrative history, checklist of signs and symptoms (including duration), list of health services used during the terminal illness, and medical evidence (if any). The history of the illness elicits an unprompted account of the trend of events that eventually led to the death. The questionnaires are age-specific; there are separate forms for neonates (0 to 28 days), children (29 days to 12 years), and adults (above 12 years). Therefore, it is important to check on the age at death of the deceased to know the appropriate questionnaire to use. The tools are widely used in most of HDSS [8,23-25]. The questionnaire used was the 2002 VA form from INDEPTH, based on the form from WHO/CDS/CSR/ISR/99.4, which has been well described [5].

A separate team of interviewers (specifically trained to conduct VAs) and administer the age-specific VA tool interview a family member who was closest to the deceased during the terminal illness and death. The interview is conducted after 40 days after the date of death to allow for the mourning period.

### Physician VA review

Each completed form is submitted to two physicians independently to ascertain the probable cause of death; in case of discordance, a third physician is invited and majority rule is applied. If the third physician determines a different cause, the case is coded as undetermined [26,27]. This is the most common method used to assign causes of death using VA [26,28-30]. Classification from the 10^th ^revision of the International Classification of Diseases (ICD-10) was used. Physicians are updated on coding with trainings conducted at least once a year. In Ifakara HDSS off-site physicians are used deliberately to avoid potential bias in coding by those who have an intimate knowledge of the population and intervention.

### VA completion rates

Four indicators are used to assess completion rates at each stage in the process in assigning causes of death: 1) number of interviews/total number of deaths identified in the community; 2) number of forms completed (i.e., deaths)/total number of forms submitted to the physicians; 3) number of deaths coded with a specific cause assigned/total number of completed forms submitted for coding; and 4) number of deaths coded with specific causes assigned/total number of forms reviewed for coding. All of the proportions are converted to percents.

### Factors associated with undetermined cause of deaths

Variables related to household compositions and socio-demographic characteristics of the respondents were included: residing with father or mother, number of deaths in the household, place of death, age category at death (neonates, children, or adult), the relationship of the respondent with the deceased, level of education of the respondent, and age and sex of the respondent.

### Data analysis and management

The data collected within the HDSS framework were used for analysis. Variables included in the analysis were extracted from different files of the Ifakara HDSS database. We performed the descriptive analysis by age and sex and by other variables, including the respondent's relationship to the deceased, the relationship to the head of household, and the place of death. The proportions were expressed as percentages and used to determine the completion rates in each step of the VA processing. All percentages refer to the preceding step in the VA processing sequence. Factors associated with undetermined cause of death were determined using a univariate logistic regression model. In order to adequately adjust for confounders, multivariate logistic regression was also used to determine association between selected independent variables and the outcome variables ("undetermined" cause of death). Two models were fitted, one for neonates and children and the other for adult deaths. Stata version 10 was used for analysis.

## Results

### Socio-demographic characteristics of the respondent

From 2002 to 2007, a total of 5,027 deaths (an average of 838 per year) were identified by the Ifakara HDSS field interviewers during the routine rounds. Of the deaths, 50% were males. The mean age at death was 31 years for the entire period of the study.

Fifty-six percent of all deaths were those aged 12 years and above. Most respondents (68%) had completed primary education, and 34% of the respondents for adult deaths were either the deceased's son or daughter. Sixty-eight percent of deaths occurred outside formal health facilities. Deaths ranged from one to four per household over the period of analysis. Over the study period, 38% of respondents were children of the heads of household. About 52% and 65% of the respondents reported residing with their mothers and fathers, respectively. Swahili was the main language used in the interviews during the VA in Ifakara HDSS (Table 1).

**Table 1:** Socio-demographic characteristics of deaths and VA respondents

	n	%
Age at death (mean)^ŧ^	31	
0 to 28 days	555	11.0
1 month to 12 years	1653	32.9
Above 12 years	2819	56.1
Sex of the deceased^ŧ^		
Female	2515	50.1
Male	2512	49.9
Respondent's level of education		
Primary	2886	68
Secondary	570	13.4
Above secondary	249	5.9
No education	539	12.7
Relationship of the respondent with the deceased*		
Partner	666	27.8
Son/daughter	817	34.1
Parent	138	5.8
Other	773	32.3
Relationship of the respondent with the deceased**		
Mother	1026	57.1
Father	498	27.8
Grandmother	149	8.3
Grandfather	28	1.6
Other	93	5.2
Relationship to the head of household		
Child	1601	37.7
Grandchild	288	6.8
Partner	421	9.9
Self	1092	25.7
Other	842	19.8
Residing with mother		
Yes	2229	52
No	2015	48
Residing with father		
Yes	2775	65.4
No	1469	34.6
Number of deaths per household		
One	2339	55.1
More than one	1905	44.9
Place of death		
Health facility	1345	31.7
Outside health facility	2899	68.3
Undetermined cause of death assigned		
Yes	3095	72.9
No	1149	27.1

^ŧ ^Includes all eligible deaths

* Adult deaths

** Neonatal and child deaths.

### Completion rates of VA

**Figure 1:**
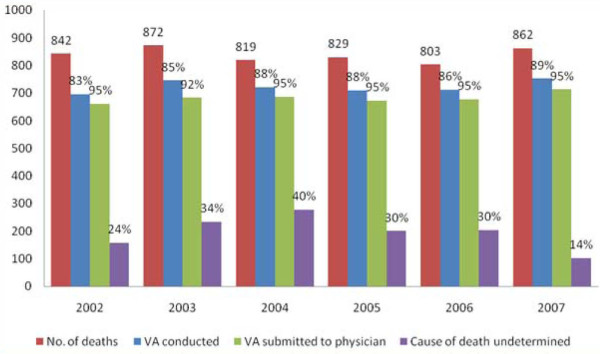
**The death distribution and completion rate in processing the VA**.

Of the deaths reported during the study, 4,244 (84%) had VA interviews conducted. The completion rate in conducting the VA over that period ranged from 83% in 2002 to 89% in 2007. Of the 4,094 VA forms submitted to physicians for ascertaining the possible cause of death, 94% ended with a cause of death specified. The coding completeness was lowest in 2003 (92%) compared to other years. There were significant differences across years in the number of deaths assigned a cause of death as undetermined (14% in 2007 and 40% in 2004) (Figure 1).

**Figure 2:**
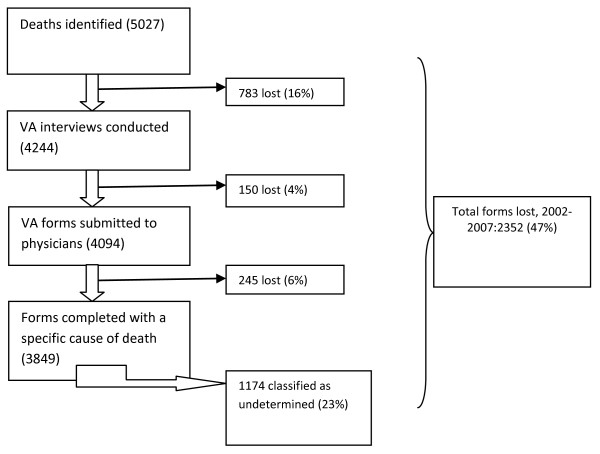
**Forms lost in processing VA at Ifakara 2002-2007**.

During the period of analysis, 16% of the deaths identified for VA were lost at the stage between community identification and VA interviews, 4% were lost between VA interviews and physicians' determination of cause of death, and 6% were lost due to logistic issues when sending forms to physicians. A total of 1,178 (23%) of the deaths identified were lost before cause of death assignment. In addition, physicians did not assign a specific cause of death (undetermined death assignment) for 1,174 respondents that were interviewed. Over the period of analysis, 2352 (47%) deaths were not assigned a specific cause, whether because they were lost or they were assigned an undetermined cause (Figure 2).

### Factors associated with undetermined cause

The current study has shown that about 31% of the death forms submitted to clinicians ended with an undetermined cause of death. For neonatal and childhood deaths, age at death, the level of education of the respondent, and place of death were associated with likelihood of undetermined cause of death. After adjusting for confounders, the neonatal and childhood deaths that occurred outside health facilities were significantly more likely, 33%, to end with undetermined cause (odds ratio [OR] = 1.33, 95% confidence interval [CI] (1.05, 1.67), p = 0.016). If the respondent had attained a secondary level of education, the death was 24% less likely to end with an undetermined cause compared with those who had no education (OR = 0.76, CI (0.60, 0.96), p = 0.023). If the respondent was related to the deceased in a way besides mother, father, or grandmother, an undetermined cause assignment was significantly more likely, but its significance disappeared after adjusting for other variables (OR = 1.57, CI (0.66, 3.77), p = 0.309).

Among adult deaths (12 years and above), the relationship with the respondent was the only variable significantly associated with undetermined cause of death. A respondent who was a child of the deceased increased the odds of the death being coded as undetermined compared to respondents that were partners (husband or wife) (OR = 1.35, CI (1.04, 1.75), p = 0.023) (Table 2).

**Table 2:** Factors associated with undetermined cause of death^a^

	Neonates and children			Adults			Neonates and children			Adults		
	OR	CI (95%)	P	OR	CI (95%)	P	OR	CI (95%)	P	OR	CI (95%)	P
Age	0.98	(0.93-1.03)	0.381	0.99	(0.99-1.00)	0.09	0.95	(0.89-1.00)	0.09	0.99	(0.99-0.99)	0.03
Sex												
Female	1											
Male	1.06	(0.85-1.32)	0.587	0.93	(0.78-1.10)	0.405	1.06	(0.85-1.32)	0.615	0.99	(0.80-1.23)	0.939
Respondent's level of education												
Primary				0.7	(0.41-1.18)	0.183				0.74	(0.46-1.34)	0.373
Secondary	0.78	(0.63-0.98)	0.035	0.87	(0.70-1.07)	0.189	0.76	(0.60-0.96)	0.023	0.88	(0.71-1.10)	0.28
No education	1											
Relationship of the respondent with the deceased*												
Partner	1											
Son/daughter	1.11	(0.89-1.40)	0.345	1.11	(0.89-1.40)	0.345				1.35	(1.04-1.75)	0.023
Parent	1.04	(0.69-1.56)	0.857	1.04	(0.69-1.56)	0.857				1.26	(0.82-1.95)	0.298
Other	1.06	(0.85-1.33)	0.597	1.06	(0.84-1.34)	0.597				1.21	(0.93-1.56)	0.147
Relationship of the respondent with the deceased* *												
Mother	1											
Father	1.11	(0.86-1.43)	0.408				1.08	(0.83-140)	0.565			
Grandmother	0.87	(0.57-1.33)	0.534				0.96	(0.60-1.53)	0.864			
Other	1.45	(0.96-2.20)	0.08				1.57	(0.66-3.77)	0.309			
Relation to the head of household												
Child	1											
Grandchild	0.92	(0.67-1.26)	0.606	0.42	(0.09-1.89)	0.251	0.92	(0.65-1.45)	0.643	0.41	(0.09-1.91)	0.259
Partner				1.41	(0.99-1.98)	0.051				1.68	(1.02-2.76)	0.04
Self				1.15	(0.84-1.56)	0.378				1.42	(0.91-2.24)	0.125
Other	0.98	(0.68-1.43)	0.929	1.09	(0.79-1.52)	0.576	0.96	(0.64-1.45)	0.862	1.3	(0.83-2.04)	0.255
Residing with mother												
No	1											
Yes	1.05	(0.61-1.82)	0.858	0.91	(0.69-1.19)	0.48	0.94	(0.50-1.74)	0.833	0.97	(0.65-1.45)	0.894
Place of death												
Health facility	1											
Outside health facility	1.31	(1.04-1.64)	0.02	0.9	(0.74-1.09)	0.302	1.33	(1.05-1.67)	0.016	0.91	(0.74-1.11)	0.342

** Neonatal and child deaths

* Adult deaths.

## Discussion

The current analysis has found that annual VA interview completion rates are high and similar with those found in other studies [8,31]. The verbal autopsy interview was completed for 84% of the deaths identified by Ifakara HDSS between 2002 and 2007. This is considered high in resource-constrained rural settings [22,32]. This achievement reflects the strength of the HDSS system in tracking vital events, the field operation, and the timing of the VA interviews. All interviews were conducted in Swahili, unlike other studies that reported language as a limit in processing the verbal autopsy [33]. For the 16% of the deaths for which VA interviews were not conducted, this was likely due to outmigration of the care takers within or soon after the 40 days of mourning. Refusal is also a potentially limiting factor for conducting the VA interviews. These factors have not been quantified in this study.

The subsequent stages in the process of assigning cause of death presented more challenges than the community identification of deaths. This poses a risk of underestimation of the burden of disease. Logistic issues preventing submission of the VA forms to physicians for coding causes a significant proportion of forms to have missing causes of death. In this study, 4% of the forms were not submitted to the physicians.

Furthermore, although the undetermined cause of death can be redistributed among the three causes as assigned by different physicians, still there is high proportion (31%) of deaths that were not assigned specific causes of death (they were coded as undetermined cause). Most of the undetermined cases were children and adults. This contradicts other studies that reported problems applying VA to neonatal deaths [17,34,35]. This observation might be due to the fact that most of the respondents to the neonatal deaths were the mothers, fathers, or grandmothers, who were likely to have good understanding of the illness and were likely very close to the deceased. These findings underscore the importance of the relationship of the deceased and the VA respondent.

A significant number of deaths occurred outside health facilities, and this underscores the continued relevance of VA in determination of cause of death in settings with inadequate vital registration systems [17]. As observed in this study, children who died outside of health facilities were more likely to be coded as undetermined. As this group is the target for VA, perhaps the tool needs to be improved further to identify the most appropriate respondent.

Another point to note is that the proportion of specific causes coded as undetermined varied significantly across years but improved markedly in 2007. This might be due to the fact that in 2007 there was more than one retraining session, unlike in other years.

The current analysis has shown the continued relevance of VA as tool for determination of cause of death in settings without or limited vital registration systems. The results raise several concerns about the continuing use of physicians in reviewing and interpreting VA data [36,37].

## Conclusion

There is high completion rate in the initial stages of VA, but a number of deaths are still lost during the later stages of VA process. The highest proportion of loss was due to physicians not assigning a definite cause of death after receiving the VA forms. Results suggest that the choice of the respondent and the location of the death have an impact on the final assignment of the cause of death across all age groups. This study has provided insight into factors affecting full coverage of verbal autopsy diagnoses and limitations to using verbal autopsy-based causes of death in SSA. Although physician review is the most commonly used method to ascertain probable causes of death, it may have limitations, and further work is needed to provide more information on the challenges faced by physicians in interpreting VA forms. There may be a need to identify alternative methods or improve physician review.

## List of abbreviations

SSA: sub-Saharan Africa; HDSS: Health and Demographic Surveillance System; VA: verbal autopsy.

## Competing interests

The authors declare that they have no competing interests.

## Authors' contributions

MAM is responsible for coordinating the HDSS, data preparation for analysis, performing all data analysis and interpretation, drafting and revising the manuscript. MAM gave final approval for submission. RN participated in coordination the HDSS and provided guidance for reviewing and data analysis. HM, AL, HU, and SA reviewed the statistical analysis and the manuscript. CM and JC participated in managing the all field data collection in the period of analysis.

All authors have read and approved the final manuscript.
